# A new species of *Amphictene* (Annelida, Pectinariidae) from the Gulf of Mexico, with a redescription of *Amphictene guatemalensis* (Nilsson, 1928)

**DOI:** 10.3897/zookeys.367.6038

**Published:** 2014-01-06

**Authors:** María E. García-Garza, J.A. de León-González

**Affiliations:** 1 Universidad Autónoma de Nuevo León, Fac. de Ciencias Biológicas, Laboratorio de Biosistemática, Av. Universidad s/n Cd. Universitaria Ap. Postal 5 ‘‘F’’, San Nicolás de los Garza, Nuevo León, C.P. 66451, México

**Keywords:** Taxonomy, polychaete, Pectinariidae, *Amphictene guatemalensis*, Gulf of Mexico

## Abstract

The genus *Amphictene* is reported for the first time from Mexico. Previous records for America are restricted to Brazil (*Amphictene catharinensis*) (Grube, 1870), and Guatemala (*Amphictene guatemalensis*) (Nilsson, 1928). In this paper we describe a new species, *Amphictene helenae* **sp. n.**, characterized by the presence of three pairs of tentacular cirri, while other species have only two pairs. The new species is closely similar to *Amphictene catharinensis*, and can be distinguished by the presence of a circular group of glandular papillae inserted between the lines of glandular cirri present from the second segment. *Amphictene guatemalensis* is redescribed based on type material; it differs from the new species in the presence of two pairs of tentacular cirri on segments 1 and 2, six pairs of glandular cirri on the third segment, and four glandular lobes fused in pairs on the fourth segment.

## Introduction

Pectinariidae de Quatrefages, 1865, comprises a group of benthic polychaetes living in characteristically shaped tubes, made of different types of materials, such as sand grains, mollusk shell fragments, foraminifers or coral fragments; the tubes resemble an “ice cream cone”. These worms live in soft bottom sediments with the cephalic region pointing downward, and posterior end upwards to the surface. This family currently contains five genera: *Amphictene* Savigny in Lamarck, 1818, *Cistenides* Malmgren, 1866, *Lagis* Malmgren, 1866, *Pectinaria*, Savigny in Lamarck, 1818 and *Petta* Malmgren, 1866. All these genera, except *Amphictene*, have been reported previously from Western Mexico. Nevertheless, Pectinariidae has not been previously reported from Eastern Mexico.

**Figure 1.**
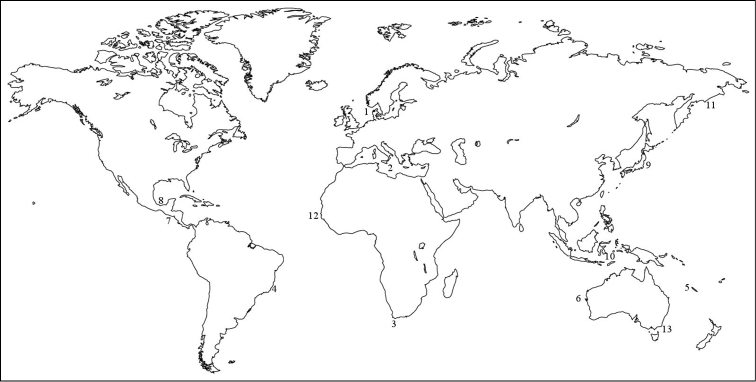
A world map showing the reported type locations of the species of *Amphictene*: **1** *Amphictene auricoma* **2** *Amphictene auricoma mediterranea* **3** *Amphictene capensis* **4** *Amphictene catharinensis* **5** *Amphictene crassa* **6** *Amphictene favona* **7** *Amphictene guatemalensis* **8*** A. helenae* **9** *Amphictene japonica* **10** *Amphictene leioscapha* **11** *Amphictene moorei* **12** *Amphictene souriei* **13** *Amphictene uniloba*.

*Amphictene* is represented by 12 species, and one subspecies (Hutchings and Peart 2002): *Amphictene auricoma* (Müller, 1776) from Denmark, *Amphictene capensis* (Pallas, 1776) from Cape of Good Hope, *Amphictene catharinensis* (Grube, 1870) from Santa Catarina Island, Brasil, *Amphictene crassa* (Grube, 1870) from New Caledonia, *Amphictene favona* Hutchings & Peart, 2002 from NSW, Australia, *Amphictene guatemalensis* (Nilsson, 1928) from Guatemala, *Amphictene japonica* (Nilsson, 1928) from Japan, *Amphictene leioscapha* (Caullery, 1944) from Banda, Indonesia, *Amphictene moorei* (Annenkova, 1929) from East coast of Siberia, *Amphictene souriei* (Fauvel, 1949) from Dakar, West Africa, *Amphictene uniloba* Hutchings & Peart, 2002 from NSW, Australia,and *Amphictene auricoma mediterranea* (Nilsson, 1928) from Mediterranean Sea (Fig. 1). In this study, a new species from the southern Gulf of Mexico is described; furthermore, *Amphictene guatemalensis* is redescribed based on type material.

## Material and methods

Type and non-type material of *Amphictene helenae* sp. n., were collected in Bahamintas Beach, Ciudad del Carmen, Campeche (18°38'36"N, 91°49'51"W). The specimens were collected by hand from the intertidal zone, in mixed sediments of coarse sand and shells fragments, using two sieves of 0.5 and 1.0 mm, at depths of 0.50–1.0 m. Specimens were fixed with 10% formalin, and preserved in ethanol 80%. The terminology used follows Hutchings and Peart (2002). The holotype was deposited in the Polychaetological Collection of the Universidad Autónoma de Nuevo León (UANL), México, and paratype were deposited in Los Angeles County Museum of Natural History, Allan Hancock Foundation (LACM-AHF), Los Angeles, USA. Specimens of *Amphictene* sp. A, are deposited in the National Museum of Natural History, Smithsonian Institution (USMN), Washington, USA.

Type material of *Amphictene guatemalensis* was borrowed from the collection of the Zoologisches Institut und Zoologisches Museum der Universität Hamburg (HZM-P), Germany.

Methyl green staining was used to determine specific patterns of glandular areas. Specimens were immersed for two minutes in a saturated solution of methyl green in 70% ethanol; later, specimens were washed with ethanol 70% to remove excess methyl green, according to Warren et al. (1994).

In this paper, we propose to use the term “bayonet shaped” to describe the notochaeta with a median broad tooth, these kind of chaetae can be observed on *Amphictene catharinensis*, *Amphictene guatemalensis*, *Amphictene capensis*, as well as on the new species here described, and on some species of *Pectinaria*. Nilsson (1928) describes these chetae as short capillary chaetae *“Kurze Kapillarborste”*; Long (1973) as *“capilaris with boss separated from shaft by incision and with tapered, narrowly blade”*; Londoño Mesa (2009) as *“notochaeta with middle tooth”*.

## Systematics

### Order TEREBELLIDA Levinsen, 1883
Family PECTINARIIDAE de Quatrefages, 1865
Genus *Amphictene* Savigny in Lamarck, 1818

**Type species.** *Amphitrite auricoma* Müller, 1776; subsequent designation by Hartman 1959.

#### Amphictene helenae sp. n.

http://zoobank.org/5752E5B9-4787-4D94-9BBC-F597566C5328

http://species-id.net/wiki/Amphictene_helenae

Figure 2

**Figure 2.**
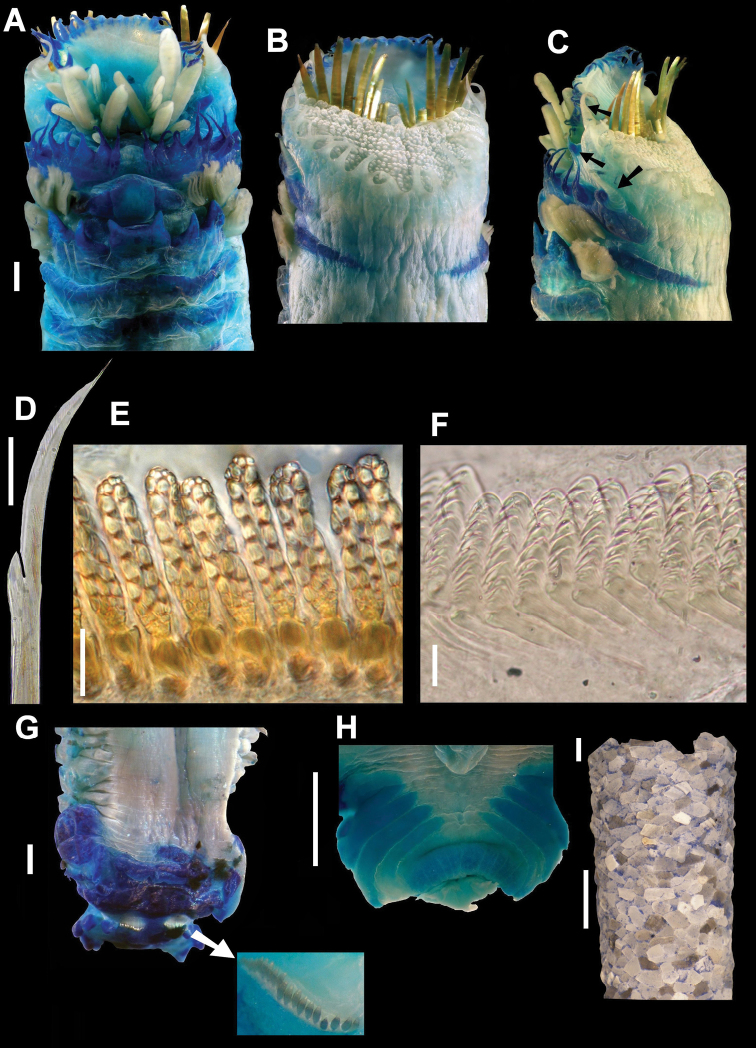
*Amphictene helenae*. Holotype. **A** ventral view of anterior end **B** dorsal view of anterior end **C** lateral view of anterior end, showing tentacular cirri **D** notochaetae from 7th chaetiger **E** front view of bayonet shaped neurochaetae from 7^th^ chaetiger **F** lateral view of neurochaetae from 7^th^ chaetiger **G** dorsal view of scaphe (**G’**) scaphal hooks detail **H** ventral view, anal papillae **I** tube of holotype. Bar scale = **A, B, C, G, H** = 1mm; **D** = 50mm **E, F** = 10mm; **I** = 3mm.

Amphictene sp. A.Wolf, 1984: 50-4-6 fig. 50-1, 2 a-j.

##### Type material.

Holotype (UANL-7824); Paratype (LACM-AHF-Poly 5741) Bahamintas Beach, Ciudad del Carmen, Campeche, México, St. 2, [18°41'60"N, 91°41'00"W], 0.50 m deep, January 3, 2011, coll. ME García-Garza and JH Landín-Delgado.

##### Additional material.

*Amphictene* sp. A (USMN 86826) St. I-4, off Port O’Connor Texas [28°22'60"N, 96°47'60"W], STOCS expedition, 10 m deep, May 1976; (USMN 86827) St. M-21, off Texas [27°53'60"N, 97°21'60"W], IXTOC expedition, 10 m deep, December 1980; (USMN 86828) St. IV 2419, off Apalachicola river, Texas [30°18'00"N, 84°08'00"W], MAFLA expedition, 10 m deep, November 1977.

##### Description.

Holotype complete, 25.7 mm long, 5.3 mm wide, with 19 segments, body robust and soft, light brown in color. Cephalic veil formed by one semicircular lobe inserted at base of paleae, forming shelf on buccal tentacles. Rim of cephalic veil with 38 long, thin cirri; each cirrus with subtriangular base, tapering toward distal end; curved toward dorsum (Fig. 2A4). Operculum covered by numerous papilliform structures randomly distributed; nine pairs of long and slightly curved paleae, sharply pointed. Opercular margin with 19 subtriangular cirri (Fig. 2B).

First segment with two pairs of tentacular cirri: first pair inserted on antero-posterior margin of segment; second pair arising from posterior middle part of segment, below first pair. Second segment with one pair of tentacular cirri inserted on lateral margin of segment, larger than cirri of first segment (Fig. 2C). Second segment dorsally indistinguishable, aerolated; with 8 pairs of thin and subtriangular ventral glandular cirri, attenuated distally; one plate with numerous small papillae in central part segment. Third segment with one middle ventral lobe and one pair of shorter lateral expansions. Fourth segment with six glandular lobes, robust and subtriangular, fused in pairs (Fig. 2A). Two pairs of latero-ventral branchiae inserted on segments 3 and 4, forming series of flat and free lamellae, fused only at base, anterior pair larger than posterior one (Fig. 2C).

Chaetigers 1–3 (segments 5–7) only with notopodia. Chaetigers 4-15 biramous with notopodia and neuropodia. Capillary notochaetae on chaetigers 1-15, some small, thinner, with external margin slightly denticulate, others bent with smooth margin, distally thin, slightly hirsute; bayonet-shaped notochaetae appear on chaetigers 4-15, with well-developed middle tooth, blade serrated throughout (Fig. 2D). Neuropodia wedge-shaped, slightly glandular, torus with numerous uncini arranged in single row. Neuropodial uncini with a group of 6-7 small apical teeth randomly placed, and two longitudinal rows, each one consisting of 5-6 larger teeth, and small group of basal teeth with undefined arrangement, decreasing in size towards base (Fig. 2E-F).

Last three posterior segments, without noto- or neurochaetae, followed by five fused segments forming scaphe, clearly separated from abdomen, wider than longer, with 21 pairs of short and thick scaphodal hooks, golden in color, with brown margins (Fig. 2G). Five marginal lobes triangular-shaped, with one fold in antero-dorsal end, and margin somewhat crenulate; anal lobe with large anal papilla and three pairs of lateral papillae, in middle dorsal region (Fig. 2H).

Tube cone-shaped, made of cemented shell fragments of similar sizes, most of them clear, with few dark fragments (Fig. 2I).

##### Remarks.

*Amphictene helenae* sp. n., is similar to *Amphictene guatemalensis* (Nilsson, 1928) and *Amphictene catharinensis* (Grube, 1870) by having a glandular cirrus on the second segment and lobes on segment 4. Nevertheless, these species differ in some morphological characters: *Amphictene helenae* sp. n., has three pairs of tentacular cirri, two pairs on the first segment, and a pair in the second one, while *Amphictene guatemalensis* and *Amphictene catharinensis* have only one pair in the first segment and a pair in the second one; *Amphictene helenae* sp. n., has eight pairs of glandular cirri on the second segment, and six robust glandular lobes on the fourth segment, subtriangular, fused in pairs. *Amphictene guatemalensis* has six pairs of glandular cirri in the second segment, two pairs of glandular subtriangular lobes in the fourth segment. *Amphictene catharinensis* has17 or 18 glandular cirri on the second segment, in the fourth a central lobe with subtriangular lateral extensions and two pairs of free subtriangular broad lobes. Furthermore, *Amphictene catharinensis* and *Amphictene helenae* can be distinguished by the presence of a circular group of small glandular papillae between both lines of glandular cirri on the ventral side of the second segment, which is absent in *Amphictene catharinensis*.

Examination of the material described by Wolf (1984) as *Amphictene* sp. A, from Florida and Texas, USA, and deposited at the USNM, indicated that it belongs to this new species, however, the specimens were found to be in poor condition.

##### Type locality.

Bahamintas Beach, Ciudad del Carmen, Campeche, México.

##### Distribution.

Gulf of Mexico.

##### Etymology.

The species is named in honour of Helena Landín García, daughter of the first author.

#### Amphictene guatemalensis

(Nilsson, 1928)

http://species-id.net/wiki/Amphictene_guatemalensis

Figure 3

Pectinaria (Amphictene) guatemalensis Nilsson, 1928: 46 fig. 14 a–f.
Amphictene guatemalensis . Hartman 1959: 479; Hutchings and Peart 2002:102; Fauchald 1977: 120.

##### Material examined.

Holotype (HZM V-1755), west coast of Central America, San José de Guatemala, Guatemala. [13°55'28"N, 90°47'25"W] coll. Captain R. Paeslin.

##### Description.

**Figure 3.**
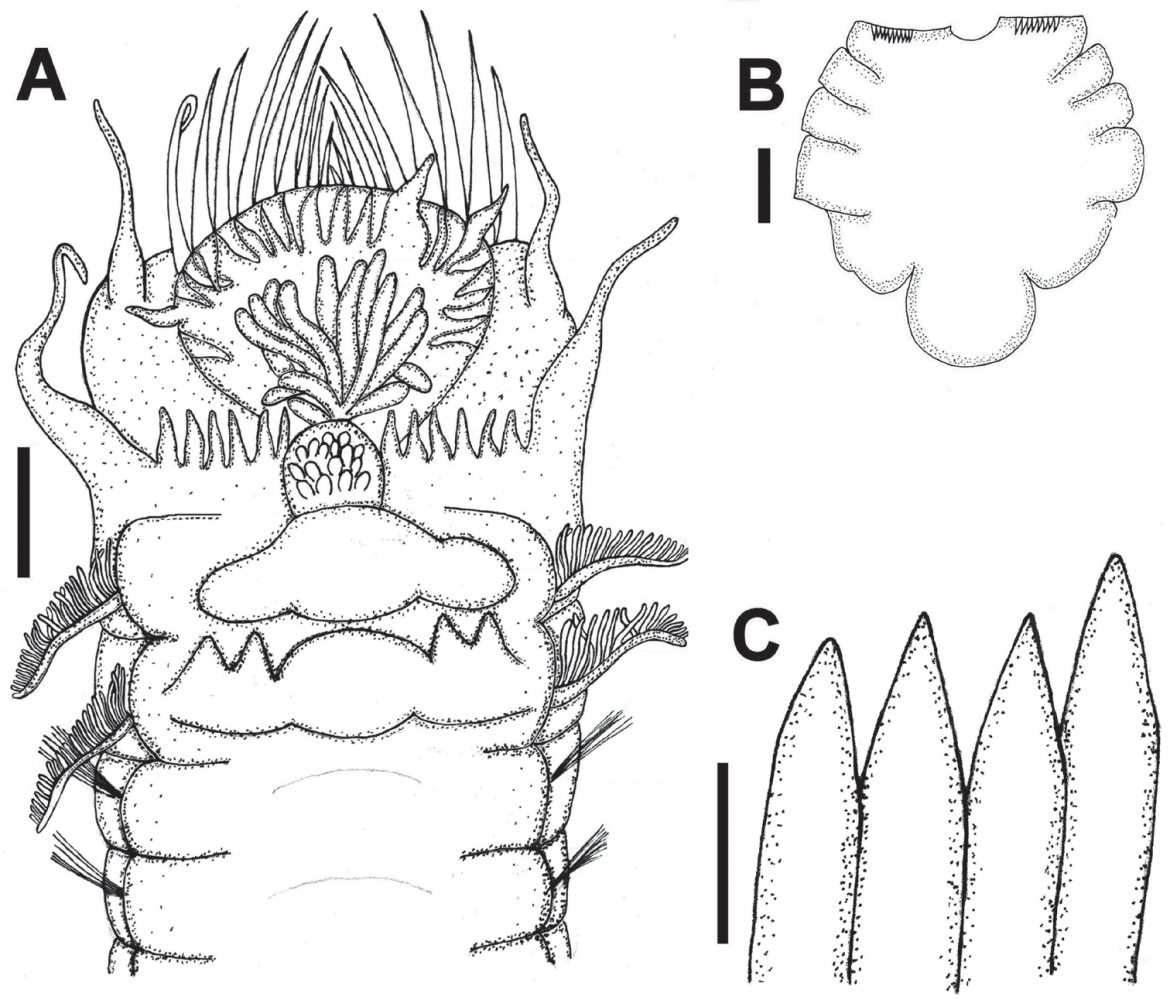
*Amphictene guatemalensis*. Holotype. **A** ventral view of anterior end **B** dorsal view of scaphe **D** scaphal hooks. Bar scale = **A, B** = 1 mm; **C** = 20mm.

Holotype complete, divided in two fragments, 14 mm long (10 mm anterior fragment, and 4 mm posterior fragment), 4 mm wide, with 19 segments. Cephalic veil formed by semicircular lobe insert at base of paleae forming shelf on buccal tentacles. Cephalic veil margin with 20 long, thin cirrus, inserted anterior end each cirrus with slightly wider base, decreasing in width distally. Opercular plate covered by numerous papilliform structures without any apparent order, with 9 pairs of long and slightly curved paleae, ending in thin filament. Opercular margin with 13 subtriangular cirri (Fig. 3A).

First pair of tentacular cirri inserted on anterior-posterior margin of first segment; second pair of tentacular cirri inserted on lateral margin of second segment. Second segment dorsally indistinguishable, very soft epithelium, with six pairs of glandular cirri on ventral side, subtriangular, fading distally, in central part of segment with a quadrangular plate having numerous small papillae. Third segment with oval central lobe, and pair of lower lateral expansions. Fourth segment with four glandular lobes, robust, subtriangular, fused in pairs, and oval central lobe. Two pairs of lateral branchiae inserted in segments 3 and 4, forming series of flat and free lamellae. Chaetigers 1-3 (segments 5–7) only with notopodia. Chaetigers 4–15 biramous with noto- and neuropodia; two types of notochaetae, thin and long simple capillary on chaetigers 1–15, and bayonet shaped ones, shorter than first ones, present on chaetigers 4–15, with well-developed median tooth and inner edge of blade dentate. Neuropodia wedge shaped, slightly glandular, torus with numerous uncini, each one with 4–5 rows of small denticles.

Last three posterior segments without notochaetae or neurochaetae, followed by 5 fused segments forming scaphe; segments broad and lobed, with smooth margin, scaphe slightly longer than wide, clearly separated from abdomen; 9 pairs of short, thick scaphal hooks, golden (Fig. 3C). Anal lobe semicircular, with cleft, bearing marginal middle papilla (Fig. 3B).

## Remarks

The holotype of *Amphictene guatemalensis* is in poor condition, broken into two parts; the epithelium is very fragile. Structures like the cirrus of cephalic veil and the glandular cirrus of second segment are bent and some others fragmented. Unlike that observed by Nilsson (1928) in the description of the species, we observed in the anal lobe only one cleft in which probably one marginal middle papilla was inserted; the remaining margin is smooth.

## Supplementary Material

XML Treatment for Amphictene helenae

XML Treatment for Amphictene guatemalensis
